# Insulin Resistance and Alzheimer’s Disease: Bioenergetic Linkages

**DOI:** 10.3389/fnagi.2017.00345

**Published:** 2017-10-31

**Authors:** Bryan J. Neth, Suzanne Craft

**Affiliations:** Department of Internal Medicine, Section on Gerontology and Geriatric Medicine, Wake Forest School of Medicine, Winston-Salem, NC, United States

**Keywords:** Alzheimer’s disease, insulin resistance, bioenergetic shift, ketone body, inflammation, metabolism, positron emission tomography

## Abstract

Metabolic dysfunction is a well-established feature of Alzheimer’s disease (AD), evidenced by brain glucose hypometabolism that can be observed potentially decades prior to the development of AD symptoms. Furthermore, there is mounting support for an association between metabolic disease and the development of AD and related dementias. Individuals with insulin resistance, type 2 diabetes mellitus (T2D), hyperlipidemia, obesity, or other metabolic disease may have increased risk for the development of AD and similar conditions, such as vascular dementia. This association may in part be due to the systemic mitochondrial dysfunction that is common to these pathologies. Accumulating evidence suggests that mitochondrial dysfunction is a significant feature of AD and may play a fundamental role in its pathogenesis. In fact, aging itself presents a unique challenge due to inherent mitochondrial dysfunction and prevalence of chronic metabolic disease. Despite the progress made in understanding the pathogenesis of AD and in the development of potential therapies, at present we remain without a disease-modifying treatment. In this review, we will discuss insulin resistance as a contributing factor to the pathogenesis of AD, as well as the metabolic and bioenergetic disruptions linking insulin resistance and AD. We will also focus on potential neuroimaging tools for the study of the metabolic dysfunction commonly seen in AD with hopes of developing therapeutic and preventative targets.

## Introduction and Overview

Alzheimer’s disease (AD) is a fatal neurodegenerative disorder that afflicts an estimated 5.3 million people in the United States. Due to socioeconomic forces such as an aging population, the prevalence of AD is projected to nearly triple to about 13.8 million Americans at an annual cost of greater than $1 trillion by 2050 (Alzheimer’s Association, 2015). AD consists of two forms; familial or early-onset AD (FAD), which constitutes less than 5% of all AD cases and is normally diagnosed prior to the age of 65 years, with clear genetic risk through inherited mutations in three main genes: amyloid precursor protein (APP), presenilin 1 (PSEN1) and presenilin 2 (PSEN2; Selkoe, 1996). Sporadic or late-onset-AD (LOAD) makes up the great majority of all AD cases and is usually diagnosed after the age of 65 years. The most prominent risk factor for the development of LOAD is advanced age and indeed the incidence of AD increases with advancing age. Although several genetic risk factors for LOAD have been identified, the most significant of these is the Apolipoprotein-E-epsilon-4 allele (APOE4), which is an isoform of the APOE gene that plays a role in cholesterol and beta-amyloid peptide (Aβ) homeostasis (Genin et al., 2011; Karch et al., 2012; Alzheimer’s Association, 2015).

AD is characterized by a progressive deterioration in cognition with significant impairments in memory, executive function and behavioral/personality changes. Neuropathologic hallmarks of AD include neuritic plaques and neurofibrillary tangles that are initially seen in the medial temporal lobes and eventually extend throughout the cortex (Braak and Braak, 1991; Braak et al., 2006). Additional misfolded proteins, including TAR DNA binding protein 43 (TDP-43) positive inclusions may be found in brains of individuals with AD (Amador-Ortiz et al., 2007). The leading hypothesis concerning the pathogenesis of AD is the Amyloid Cascade Hypothesis, which postulates that Aβ plays a central role in AD pathology leading to oxidative injury, synaptic/neuronal dysfunction and eventual neurodegeneration (Hardy and Higgins, 1992; Hardy and Selkoe, 2002). The clinical path of AD may be viewed as a continuum with an extended asymptomatic stage without cognitive or behavioral symptoms, but with documentable changes in brain pathological processes—with ultimate progression to mild cognitive impairment (MCI) and eventually dementia with cognitive and functional decline (Sperling et al., 2011).

Central metabolic dysfunction is a well-established feature of AD, evidenced by brain glucose hypometabolism that can be observed potentially decades prior to the development of AD symptoms (Reiman et al., 1996; Small et al., 2000; Sperling et al., 2011). Furthermore, there is mounting support for an association between metabolic disease and the development of AD and related dementias (Whitmer et al., 2008; Craft, 2009). Individuals with insulin resistance, type 2 diabetes mellitus (T2D), hyperlipidemia, obesity, or other metabolic disease may have increased risk for the development of AD and similar conditions, such as vascular dementia (Craft, 2009; Di Paolo and Kim, 2011). This association may in part be due to the systemic mitochondrial dysfunction that is common to these pathologies (Lesnefsky et al., 2001; Lowell and Shulman, 2005; Madamanchi and Runge, 2007; Johri and Beal, 2012). Accumulating evidence suggests that mitochondrial dysfunction is a significant feature of AD and may play a fundamental role in its pathogenesis (Yao et al., 2010; Yao and Brinton, 2011; Chaturvedi and Beal, 2013). In fact, aging itself presents a unique challenge due to inherent mitochondrial dysfunction and prevalence of chronic metabolic disease (Mammucari and Rizzuto, 2010; Cui et al., 2012).

Despite the progress made in understanding the pathogenesis of AD and in the development of potential therapies, at present we remain without a disease-modifying treatment (Armstrong, 2011; Schneider et al., 2011a,b; Howard et al., 2012; Doody et al., 2014; Salloway et al., 2014). In this review, we will discuss insulin resistance as a contributing factor to the pathogenesis of AD, as well as the metabolic and bioenergetic disruptions linking insulin resistance and AD. We will close with a review of potential tools for the study of the metabolic dysfunction commonly seen in AD.

## Metabolic Pathways to Alzheimer’s Disease: Insulin Resistance

### Overview of Insulin in the Brain

Insulin is a peptide hormone secreted principally by pancreatic beta cells with well-characterized functions in glucose/lipid metabolism, vascular regulation, and cell growth (Saltiel and Kahn, 2001; Muniyappa et al., 2007). Mounting evidence suggests that insulin plays a vital role in the central nervous system (CNS; Craft and Watson, 2004; Ketterer et al., 2011; Liu et al., 2011; Banks et al., 2012; Correia et al., 2012; Duarte et al., 2012; Cholerton et al., 2013; Craft et al., 2013; Blázquez et al., 2014). Insulin readily crosses the Blood Brain Barrier (BBB) through a saturable, receptor-mediated process (Baskin et al., 1987; Baura et al., 1993; Banks et al., 1997a,b; Woods et al., 2003). Moreover, several regions in the brain (hypothalamus, choroid plexus, etc.) may serve as a more rapid site of entry for peripheral insulin into the CNS (Baskin et al., 1987). There is continuing debate concerning production of insulin within CNS. Studies in animal models have described presence of insulin mRNA in various brain regions (Banks et al., 2012; Duarte et al., 2012). Clinical studies have described the presence of C-peptide, which is secreted at the time of insulin production in pancreatic beta cells, in cerebrospinal fluid (CSF; Ghasemi et al., 2013b; Blázquez et al., 2014). However, this too may be from the pancreas and not produced within the CNS.

Insulin exerts its action through binding to the insulin receptor (IR) with two different isoforms (IR-A and IR-B). IR-A is found in the adult nervous system and has a higher affinity for insulin than IR-B, which is found mainly is adipose tissue, hepatic tissue, and skeletal muscle (Zhao W.-Q. et al., 2004; Dou et al., 2005; Watson and Craft, 2006; Banks et al., 2012; Ghasemi et al., 2013b). However, a recent study has reported IR-B expression in astrocytes (Garwood et al., 2015), with a potential role in mediating insulin and insulin-like growth factor (IGF) function in the CNS. It is important to note that insulin-like growth factor-1 and 2 (IGF-1, IGF-2) can also bind at the IR, but at decreased affinity than insulin (Banks et al., 2012; Kleinridders et al., 2014). IRs are tyrosine kinases with alpha and beta subunits. Once insulin or other substrates are bound, the alpha subunit promotes autophosphorylation of tyrosine residues on the beta subunits leading to the recruitment of scaffolding proteins, mainly IR substrates 1 and 2 (IRS-1 and IRS-2). IRS-1, 2 ultimately connect insulin to two significant signal transduction pathways: the PI3K/Akt pathway, largely responsible for metabolic effects, lipid/protein synthesis and the Ras/ERK pathway which modulates cell growth, survival, and gene expression (De Felice and Ferreira, 2014; Kleinridders et al., 2014). IRs are located in both neurons and glia (Abbott et al., 1999). These receptors are selectively distributed throughout the brain, with higher concentrations in the olfactory bulb, cerebral cortex, hippocampus, hypothalamus, amygdala, and septum—regions of strategic importance for feeding and cognition (Havrankova et al., 1978a,b; Baskin et al., 1987; Unger et al., 1991). Similar to IR, IGF-1 receptors (IGF1-R) are also tyrosine kinases binding IGF-1/2 and insulin. Interestingly, both IR and IGF1-R can form hybrids of either heterodimers or homodimers with each other. The ultimate downstream effects depend on the ligand and type/location of receptor. Likewise, the affinity of the ligand also depends on the receptor type. The distribution of IR, IGF1-R, and their hybrids are regionally specific within the CNS (Kleinridders, 2016; Cai et al., 2017). Refer to a recent review by Kleinridders (2016) for a more comprehensive understanding of IR and IGF-1 receptors in the brain.

### Insulin and Cognition

An acute elevation of peripheral insulin (generally in response to an increase in exogenous or endogenous glucose) promotes insulin transport across the BBB into the CNS, thus facilitating its role for a variety of important brain functions (Woods et al., 2003; Kleinridders et al., 2014). The regional localization of IR in the hippocampus (Werther et al., 1987) suggests that insulin may influence memory, one of the main tasks supported by the hippocampus and closely connected structures. Intracerebroventricular administration of insulin in rats has been shown to improve passive avoidance and spatial memory (Park et al., 2000; Haj-ali et al., 2009). Clinical studies utilizing acute intravenous administration of insulin with maintenance of euglycemia have described enhanced performance in verbal memory (Craft et al., 1996, 1999, 2003; Kern et al., 2001). Similar memory improvement has been observed following administration of intranasal insulin (Benedict et al., 2004, 2008; Stockhorst et al., 2004; Reger et al., 2006, 2008; Craft et al., 2012; Schiöth et al., 2012; Claxton et al., 2013, 2015; Freiherr et al., 2013; Novak et al., 2014); these studies will be reviewed in detail below. In total, the above work forms a strong foundation for insulin’s role in memory, one of the key cognitive domains affected by AD.

Intriguingly, the process of learning may modify IR expression and function throughout specific brain regions (Zhao et al., 1999; Zhao W.-Q. et al., 2004; Agrawal et al., 2011). In rats, spatial memory training has been shown to upregulate IR mRNA in the hippocampal CA1 region and dentate gyrus and lead to increased accumulation of IR protein within the hippocampus. Moreover, training increased insulin-stimulated tyrosine phosphorylation of the IR *in vitro* in trained animals (Zhao et al., 1999). These results suggest that learning itself may influence both IR concentration and insulin signaling in the hippocampus and potentially other brain regions. It is likely that insulin plays a key role in learning and memory given IR localization in the hippocampus, IR changes in the hippocampus secondary to spatial learning, and improvements in memory secondary to insulin administration in both animal models and human studies. Although it is not entirely clear how insulin exerts its action on cognition, several mechanisms likely contribute.

### Insulin and Cerebral Glucose Metabolism

One such mechanism by which insulin may influence cognition is by affecting cerebral energy metabolism. The importance of glucose in the CNS is demonstrated by the disproportionate metabolic rate of the brain relative most organs and tissues. While the brain only makes up 2% of the average body weight, it utilizes about 25% of the body’s glucose and 20% of the body’s oxygen to meet metabolic demand (Attwell and Laughlin, 2001; Bélanger et al., 2011). The energy derived from glucose metabolism is used to maintain neuronal ion gradients and cell membrane lipid remodeling, among other processes (Attwell and Laughlin, 2001). Insulin undoubtedly is crucial for peripheral energy metabolism in adipose tissue, hepatic tissue, and skeletal muscle (Saltiel and Kahn, 2001). Until recently brain (glucose) metabolism has largely been thought of as insulin-independent. Yet, recent research has highlighted an important role of insulin in cerebral/peripheral metabolism and other functions (Banks et al., 2012; Blázquez et al., 2014).

Bingham et al. (2002) reported increased cerebral glucose metabolism after restoration of basal levels of insulin in metabolically healthy participants. Metabolic changes were most apparent in cortical areas. This work indicates that normal basal levels of peripheral insulin may play an important role in the maintenance of cerebral glucose metabolism. In a recent study of adults with metabolic dysfunction, Hirvonen et al. (2011) reported increased glucose metabolism on ^18^F-fluorodeoxyglucose (FDG) Positron Emission Tomography (PET) imaging after a hyperinsulinemic clamp procedure. Interestingly, glucose metabolism was not affected by the hyperinsulinemic condition in metabolically healthy participants (Hirvonen et al., 2011). These results support the view that insulin may modify cerebral glucose metabolism, and that effects may differ depending upon metabolic status.

Insulin likely exerts regional effects on cerebral glucose metabolism due to the localized distribution of glucose transporters (GLUTs; Schulingkamp et al., 2000; Reagan et al., 2001). A recent review by Shah et al. (2012) discusses the role of these transporters in brain disease, with a focus on AD and diabetes. The insulin independent GLUT1 and GLUT3 were traditionally believed to be the sole cerebral GLUTs (Lund-Andersen, 1979). These GLUTs are expressed at the BBB and within neurons and glia (Devraj et al., 2011; Shah et al., 2012). However, it is now apparent that insulin-responsive GLUTs, such as GLUT4 and GLUT8, are also localized within specific brain regions such as the hippocampus, cerebellum, sensorimotor cortex, hypothalamus and pituitary (Brant et al., 1993; Livingstone et al., 1995; El Messari et al., 1998; Apelt et al., 1999; Reagan et al., 2001; Shah et al., 2012). Insulin has been reported to increase cerebral GLUT4 expression and translocation (Piroli et al., 2007). Importantly, insulin-responsive GLUT4 and GLUT8 are co-localized to regions that express IR and insulin (Apelt et al., 1999; Schulingkamp et al., 2000). Our understanding of cerebral glucose metabolism continues to expand, with new insights supporting the role of insulin in mediating at least some portion of cerebral glucose metabolism, which likely impacts cognitive processes including learning and memory.

### Other Actions

In addition to insulin’s function in cerebral glucose metabolism, another mechanism by which it may impact the brain is its influence on long-term potentiation (LTP; Zhao and Alkon, 2001). In particular, insulin has been reported to affect the expression of N-methyl-D-aspartate (NMDA) receptors (Skeberdis et al., 2001). Furthermore, insulin has been shown to modulate levels of the neurotransmitters, acetylcholine and norepinephrine, which have been shown to influence cognition (Figlewicz et al., 1993; Kopf and Baratti, 1999). Insulin also serves other important functions through actions in the brain such as neuroprotective effects as well as mediation of vascular function through nitric oxide (NO) and endothelin-1 (Banks et al., 2012; Katakam et al., 2012; Blázquez et al., 2014). The importance of insulin’s role in the CNS is becoming increasingly clear as converging evidence demonstrates that disrupted insulin signaling (insulin resistance) may promote neurodegenerative disorders, such as AD (Craft, 2007, 2009; Neumann et al., 2008; Cholerton et al., 2011; Correia et al., 2012; Chen and Zhong, 2013; Ghasemi et al., 2013b; Blázquez et al., 2014; De Felice and Ferreira, 2014; Sridhar et al., 2015).

### Overview of Insulin Resistance

Insulin resistance occurs when insulin binding to its receptors has diminished effects. Although this term is most commonly applied to decreased glucose clearance from the blood and entry into target tissues (Reaven, 1983, 2003; DeFronzo et al., 1992), it can also refer to insulin’s ability to engage its canonical signaling network in any target tissue. In the periphery this resistance is accompanied by increased release of insulin from the pancreas to meet the demand of chronically elevated levels of glucose and/or increased amount of adipose tissue that requires insulin for its glucose metabolism (DeFronzo et al., 1992). Prolonged elevation of systemic insulin may ultimately lead to a dysfunction in insulin signaling (DeFronzo, 2010). This chronic elevation in peripheral insulin levels also impacts central insulin availability and function. Insulin’s passage through the BBB is transporter-mediated (Banks et al., 2012). In a healthy state, an acute, transient rise in peripheral insulin leads to an increase in CNS insulin, where it enters the brain. Chronic peripheral hyperinsulinemia leads to the downregulation of insulin transporters at the BBB, which in turn decreases the amount of insulin that may enter brain (Wallum et al., 1987; Schwartz et al., 1990; Banks et al., 2012). This CNS insulin deficiency may potentially lead to impairments in memory, neuroprotective effects, synaptic transmission, as well as likely contributing to the development of neurodegenerative disease (Craft and Watson, 2004; Craft, 2007; Cholerton et al., 2011; Correia et al., 2012; Ghasemi et al., 2013a; Blázquez et al., 2014; De Felice and Ferreira, 2014; De Felice and Lourenco, 2015). Importantly, negative impacts of insulin resistance occur years prior to the development of clinically defined diabetes (Roriz-Filho et al., 2009). Early defects in insulin signaling may be associated with pathologic brain changes even decades before clinical symptoms of the disease (Roriz-Filho et al., 2009). Moreover, patients may not appreciate significant symptoms until the disease process has already exerted a negative, and potentially irreversible impact on peripheral tissues and the brain (Sperling et al., 2011; Sridhar et al., 2015).

### Impact of Insulin Resistance on the Brain

In a metabolically healthy state, an acute elevation of insulin levels has a beneficial impact on cognitive function. However, chronically elevated insulin greatly diminishes insulin’s end-organ effects (Neumann et al., 2008). Evidence supporting this include impaired learning in animal models of T2D and in humans with the disorder (Greenwood and Winocur, 2001). Vanhanen et al. (1998) described lower scores on the Bushcke Selective Reminding Task (verbal learning and memory) in older adults with impaired glucose tolerance. Insulin resistance also impacts brain structure and function corresponding with changes in brain volumes and cerebral glucose metabolism. Convit et al. (2003) described lower hippocampal volumes in older adults with impaired glucose tolerance, which were also associated with lower scores on delayed recall of a logical memory task. Similarly, a study by Kerti et al. (2013) reported that adults with higher fasting glucose and HbA1c had lower delayed recall, learning, and memory consolidation utilizing the Rey Auditory Verbal Learning Test. Higher fasting glucose and HbA1c also correlated with lower hippocampal volume and altered hippocampal microstructure as determined by Mean Diffusivity, a Diffusion Tensor Imaging (DTI) metric (Kerti et al., 2013). Further analysis in this group suggested that the beneficial effects of lower blood glucose on learning and memory could in part be explained by the hippocampal changes described in the study (Kerti et al., 2013). Moreover, we have previously reported that older adults with insulin resistance (pre-diabetes or diabetes without treatment) showed AD-like patterns of reduced brain glucose metabolism, as quantified with FDG PET imaging. Glucose hypometabolism was most apparent in frontal, parietotemporal, and cingulate cortices (Baker et al., 2011). This finding is profound in that it provides evidence that insulin resistance affects similar brain areas as AD, supporting the view that insulin resistance may promote neurodegenerative disease. Diabetes has been shown to be a strong predictor of cognitive decline in older adults (Yaffe et al., 2004, 2006; Cheng et al., 2012; Biessels et al., 2014). In fact, those with diabetes may be twice as likely to experience a decline in cognition over 5 years relative to those without the disorder (Tilvis et al., 2004). Likewise, several epidemiologic studies have described an association between insulin resistance and cognitive impairment and/or the development of dementia in older adults (Hassing et al., 2004; Yaffe et al., 2004; Strachan, 2011; Cheng et al., 2012). Cognitive impairment is not restricted to changes in learning and memory, but also other domains. For example, Abbatecola et al. (2004) showed that insulin resistance was associated with longer time to complete Trail Making Test—Part B. The Trail Making Test is a task of processing speed, cognitive flexibility and visual motor skills and has sensitivity for a variety of disorders negatively affecting cognition (Bowie and Harvey, 2006). Given current evidence insulin resistance must be considered an important risk factor for cognitive decline.

Research in humans is largely supported by work in animal models of insulin resistance, describing its negative impact on cognitive performance. Stranahan et al. (2008) demonstrated that a high-saturated fat diet supplemented with high fructose corn syrup-laden water, as a proxy for a “Western Diet,” led to worse performance on a spatial memory (water-maze) task relative to control animals and reduced LTP after 8 months on diet. Rats on a high saturated fat diet developed insulin resistance, which was accompanied by lower concentrations of hippocampal brain-derived neurotrophic factor (BDNF; Stranahan et al., 2008). These results provide evidence supporting the profound impact that insulin resistance may have on the mammalian brain, an in particular on the hippocampus, one of the primary brain regions implicated in AD pathology.

In summary, work in both humans and animal models suggest that insulin resistance has detrimental effects on cognition, most notably learning and memory. Given the vital influence of insulin resistance on brain, it is important to further understand the metabolic pathways that may be impacted in such conditions, as well as how these pathways related to AD and other neurodegenerative disorders.

## Bioenergetic Disruptions in Insulin Resistant State Relevant to Alzheimer’s Disease

Although the pathogenesis of insulin resistance and AD are yet to be fully elucidated, both share common pathologic features, supporting the notion that systemic insulin resistance may ultimately promote AD. Common features of an insulin resistant state and AD include inflammation, dyslipidemia, amyloidogenesis and overt bioenergetic dysfunction.

### Inflammation and Vascular Dysfunction

Chronic inflammation is detrimental to the body and brain (Blasko et al., 2004; Khansari et al., 2009; Schwartz and Baruch, 2014), with elevated levels of inflammatory cytokines and chemokines reported in AD and insulin resistance (Blum-Degen et al., 1995; Pradhan et al., 2001; Kubaszek et al., 2003; Swardfager et al., 2010). Not surprisingly, chronic low-level inflammation in adipose tissue has been hypothesized to contribute to the pathogenesis of insulin resistance (Festa et al., 2000; Akash et al., 2013). A type of inflammatory cell, the adipose tissue macrophage, may be a central player in perpetuating the inflammatory cascade that ultimately leads to insulin resistance and T2D (Lee, 2013). Inflammation has been a feature commonly identified in studies of AD and other neurodegenerative disorders (Akiyama et al., 2000; Amor et al., 2010; Wyss-Coray and Rogers, 2012). Inflammatory cytokines are commonly elevated in the plasma and CSF of Alzheimer’s patients (Blum-Degen et al., 1995; Swardfager et al., 2010). Furthermore, research has explored the role of other inflammatory mediators in AD with focus on BBB and vascular integrity (Ryu and McLarnon, 2009; Takeda et al., 2013).

A powerful mediator of inflammation and vascular dysfunction common to both insulin resistance and AD are advanced glycation end products (AGEs), which are glycated proteins and lipids formed through non-enzymatic glycosylation after exposure to glucose (Singh et al., 2001). There are increased AGEs in adults with insulin resistance and diabetes relative to healthy controls (Goldin et al., 2006; Unoki et al., 2007; Unoki and Yamagishi, 2008; Yamagishi et al., 2012). Accumulation of AGEs may exert negative effects on tissues. For example, AGE binding to its target, the receptor for advanced glycation end products (RAGE), promotes upregulation of nuclear factor-kappa beta (NF-κβ), which is a transcription factor and crucial mediator of inflammation (Goldin et al., 2006). AGEs may block the production of NO in the endothelium, thereby mitigating its vasodilatory effects. Moreover, a complex of AGE-RAGE may interfere with vascular structure, making it permeable to macromolecule invasion and resultant pathology (Goldin et al., 2006). Wautier et al. (1996) reported that inhibition of RAGE prevents the AGE-related changes in vascular permeability within diabetic rats. This research suggests that a prominent feature in individuals with insulin resistance (AGE) may propagate an inflammatory cascade and vascular injury. AGEs are also more elevated in adults with AD than age-matched controls and may ultimately contribute to AD pathology (Sasaki et al., 1998; Srikanth et al., 2011). AGEs have been discovered in amyloid-containing senile plaques, tau-containing neurofibrillary tangles, neurons and glia (Lüth et al., 2005). Glycation of Aβ has been shown to enhance its aggregation (Sasaki et al., 1998) AGEs have been reported to stimulate tau hyperphosphorylation, which is attenuated with inhibition of RAGE in rats (Lüth et al., 2005). Moreover, AGEs stimulate similar oxidative stress, inflammation, and vascular pathology in the brain as that found in the periphery (Ramasamy et al., 2005).

### Dyslipidemia

Insulin is an important mediator of lipid metabolism, and a core feature of insulin resistance is dyslipidemia (DeFronzo and Ferrannini, 1991; Savage et al., 2007). Lipids and cholesterol constitute a significant portion of the brain mass, with continual turnover, especially at synapses and cellular connections (Robinson et al., 1992; Purdon et al., 2002). Impaired lipid metabolism may therefore have a profound impact on the brain and contribute to neurologic disease. The characteristic lipid profile of chronic insulin resistance includes elevated free fatty acids (FFA), which inhibit the insulin-related suppression of very low density lipoprotein (VLDL) secretion by the liver (DeFronzo and Ferrannini, 1991). This contributes to an altered lipid balance with elevated VLDL and other lipids, which may perpetuate an insulin resistant state (DeFronzo and Ferrannini, 1991; Kamagate et al., 2008). Higher low density lipoprotein (LDL) and lower high density lipoprotein (HDL) levels are known cardiovascular risk factors, and could play a role in development of AD-related amyloid deposition, potentially due to the impact of cholesterol on Aβ processing within the brain (Reitz, 2013; Berti et al., 2015).

Furthermore, various genetic studies including genome-wide association studies (GWAS) have identified several genes involved with lipid and cholesterol metabolism as increasing risk for AD. Most notably is Apolipoprotein-E (APOE), followed by Apolipoprotein-J (APOJ or Clusterin, CLU), ATP-binding cassette subfamily A member 7 (ABCA7) and sortilin-like receptor (SORL1; Reitz, 2013). Although our understanding of dyslipidemia, cholesterol metabolism and its relation to AD is incomplete, it seems plausible that altered states of systemic lipid metabolism may contribute to pathologic brain changes seen in the disease. A study by Reed et al. (2014) reported that higher levels of LDL and lower levels of HDL were associated with higher amount of amyloid concentration as determined by Pittsburgh Compound B (PiB) PET imaging. These results suggest a potential association between dyslipidemia and cardiovascular risk and the accumulation of cerebral amyloid, which may in turn be mediated by insulin resistance as well as other causes of disturbed lipid metabolism, such as carriage of the APOE4 allele. Intriguingly, a recent study suggests that carriage of an APOE4 allele may contribute to diminished IR signaling by directly interacting with the IR, impairing its trafficking and ultimately leading to the IR being trapped within endosomes (Zhao et al., 2017). This may provide novel insight into the role of APOE and its relation to insulin signaling, thus explaining the differential response to intranasal insulin in APOE4 carriers (Reger et al., 2006; Claxton et al., 2013, 2015).

### Amyloidogenesis

Both AD and T2D are amyloidogenic conditions; Aβ (1–40 and 1–42) is found at increased concentrations in AD and amylin (islet amyloid polypeptide, IAPP) is found at elevated levels within insulin resistant states (Cooper et al., 1989; Hardy and Higgins, 1992; Lim et al., 2010). Amylin accumulates primarily in the pancreas, which may potentiate the development of T2D and worsening insulin resistance as pancreatic beta cells become depleted (Cooper et al., 1989; Jackson et al., 2013). Recent studies have also described amylin accumulation in other tissues throughout the body, including in cerebral vasculature and brain parenchyma (Despa et al., 2012; Jackson et al., 2013; Srodulski et al., 2014). Jackson et al. (2013) described the accumulation of oligomeric amylin and amylin plaques in the temporal lobes, vasculature and perivascular spaces of older adults with T2D, but not in age-matched controls. A similar pattern of amylin deposition was found in the cerebral vessels and brain parenchyma of adults with AD, even in the absence of T2D. Co-localized amylin and Aβ deposition were also observed (Jackson et al., 2013). These results suggest that amylin may have a pathologic impact on the brain and contribute to metabolic risk for AD. It is becoming increasingly clear that the amylin and Aβ may not be mutually exclusive. Research suggests that amylin may indeed contribute to the accumulation of Aβ in AD through seeding effects (Yan et al., 2014; Oskarsson et al., 2015). Yet, further research into this Aβ-amylin interaction must be performed in order to fully understand the molecular link between these two amyloidogenic molecules.

Furthermore, insulin has been shown to impact Aβ, which has traditionally been implicated in the pathogenesis of AD (Hardy and Higgins, 1992; Hardy and Selkoe, 2002). Evidence in animal models suggests that brain insulin deficiency may lead to increased formation of Aβ due to the upregulation of APP and beta-secretase 1 (BACE1), which are involved in formation of Aβ (Devi et al., 2012). *In vitro* studies have reported that insulin impacts amount of Aβ. For example, in neuronal cultures insulin stimulates the release of intracellular Aβ (1–40 and 1–42) into the extracellular space. Trafficking of intracellular Aβ and APP is accelerated as they are transported from the Golgi/trans-Golgi network to the plasma membrane (Gasparini et al., 2001). An insulin resistant state would interfere with this action of insulin and reduce trafficking of Aβ out of the cell.

A significant factor in Aβ degradation is the metalloprotease insulin-degrading enzyme (IDE; Cook et al., 2003; Farris et al., 2003; Zhao L. et al., 2004). IDE is highly expressed in tissue throughout the body, including brain, liver, skeletal muscle and kidney (Authier et al., 1996). Importantly, IDE has been described to facilitate the breakdown of both insulin and Aβ (Kurochkin and Goto, 1994; McDermott and Gibson, 1997; Qiu et al., 1998; Sudoh et al., 2002; Zhao L. et al., 2004). The IDE-dependent degradation of Aβ has been shown to occur through a PI3K-dependent mechanism (Zhao L. et al., 2004). Thus, an early insulin-resistant state (with higher levels of circulating insulin) may contribute to the accumulation of Aβ due to competition for IDE (Qiu et al., 1998; Roriz-Filho et al., 2009; Bosco et al., 2011). Research in AD mouse models has further solidified the role of insulin resistance in IDE-related amyloid pathology. Tg2576 mice with diet-induced insulin resistance had increased production of Aβ (1–40 and 1–42) in the brain relative to Tg2576 mice without insulin resistance. Interestingly, these results were associated with an increased gamma-secretase activity and decreased IDE activity (Ho et al., 2004). Moreover, IDE knockout mice have been shown to have a reduced breakdown of cerebral Aβ and insulin (Pérez et al., 2000; Cook et al., 2003; Farris et al., 2003). These results suggest that insulin resistance may independently contribute to amyloid production and exacerbate an already amyloidogenic state, such as AD.

Chronically elevated peripheral insulin may lead to lower levels of insulin within the CNS (Wallum et al., 1987; Schwartz et al., 1990; Banks et al., 2012). Initially cerebral levels of insulin may be increased (as with the periphery), yet insulin eventually decreases as BBB transport is reduced and amyloid accumulates, promoting central insulin resistance (Banks et al., 1997b). This process may negatively impact insulin’s functions in the brain and the clearance of Aβ. Patients with AD have been shown to have lower insulin in the CSF, elevated insulin in the blood, and a lower CSF:Plasma insulin ratio relative to healthy controls (Craft et al., 1998). Furthermore, increased peripheral insulin concentration may interrupt breakdown of Aβ after being transported out of the brain as well as by interfering with its exit from the brain. A potential mediator of these effects is LDL receptor-related protein (LRP-1). Tamaki et al. (2007) reported that insulin mediates the hepatic uptake of circulating Aβ (1–40) from the blood by increasing LRP-1 expression on hepatic cellular plasma membranes. This process is dose-dependent and reversed with administration of an LRP-1 inhibitor (Tamaki et al., 2007). LRP-1 has also been shown to contribute to Aβ (1–40) transport across the BBB to the peripheral circulation (Ito et al., 2006). These results suggest that insulin resistance may contribute to the accumulation of amyloid species due to impaired clearance from the brain to the periphery where it may be cleared by the liver. Both decreased removal of Aβ from the CNS and reduced degradation of Aβ once it reaches the periphery may contribute to the clogging of a peripheral Aβ “sink.” Thus, the pattern of high peripheral insulin levels and low brain insulin levels may increase risk for AD and ultimately help exacerbate disease pathology.

Additional mechanisms may relate insulin resistance to AD pathology. For example, the soluble form of Aβ is able to bind to the IR, and has been shown to disrupt insulin signaling and activation of three kinases in primary hippocampal neurons (Townsend et al., 2007). Specifically, Aβ was shown to act through diminishing insulin-induced autophosphorylation (Townsend et al., 2007). Moreover, a study by Zhang et al. (2013) suggests that Aβ promotes hepatic insulin resistance through JAK2 signaling. Taken together these results infer that over accumulation of Aβ may promote impaired insulin signaling that could further propagate disease pathology. Synaptotoxic effects also link Aβ and insulin. Synapse loss is an early event in AD pathology, which may be promoted by soluble Aβ oligomers (Selkoe, 2002; Scheff et al., 2006). Insulin has been shown to prevent binding of Aβ to synapses, thus minimizing synaptic damage (De Felice et al., 2009). Insulin has also been shown to diminish formation of Aβ oligomers, which is likely protective against Aβ oligomer-related damage (Lee et al., 2009). This research suggests that a pathologic hallmark of AD initiated by Aβ oligomers may be mitigated by insulin administration. Inflammation, dyslipidemia, and amyloidogenesis are each prominent features linking insulin resistance and AD. Yet, one of the most important connections between the two pathologies is the inherent bioenergetic dysfunction that is common to each.

### Bioenergetic Dysfunction in Insulin Resistance and Alzheimer’s Disease

Another common feature of AD and insulin resistance is mitochondrial dysfunction, which is supported by several key findings (Lowell and Shulman, 2005; Kim et al., 2008; Szendroedi et al., 2011; Johri and Beal, 2012; Chaturvedi and Beal, 2013). First, enzymes involved in energy metabolism are differentially regulated in AD. Lower activity of the pyruvate dehydrogenase (PDH) and alpha-ketoglutarate dehydrogenase (AKGDH) complexes has been reported in AD (Park et al., 1999; Blass, 2000; Starkov et al., 2004). These enzymes are fundamental for cellular respiration, with PDH being particularly important for linking glycolytic metabolism to the Kreb’s cycle. Like many bioenergetic enzymes, both may be affected by the accumulation of oxidative moieties leading to decreased enzyme efficiency and ultimate production of reactive oxygen species (ROS) and further oxidative stress (Wei and Lee, 2002).

Second, mitochondrial function in adults with AD may be affected by the accumulation of Aβ, which seems to be at least partly mediated by Aβ-binding-alcohol-dehydrogenase (ABAD; Yao et al., 2011; Chaturvedi and Beal, 2013). Specifically, Aβ has been shown to inhibit activity of complexes II and IV of the electron transport chain (ETC) and lead to increased production of ROS (Swerdlow et al., 2010; Yao et al., 2011; Chaturvedi and Beal, 2013). Reduced activity of complex IV, also known as cytochrome c oxidase (COX), has been widely reported in platelets as well as within the brain, which supports the view of AD being a systemic disorder (Parker et al., 1990; Cardoso et al., 2004). Aβ and APP have been implicated in the disruption of mitochondrial dynamics (fission/fusion), contributing to the mitochondrial dysfunction seen in AD (Wang et al., 2008, 2009; Chaturvedi and Beal, 2013).

Additionally, Aβ has been shown to impair mitochondrial calcium homeostasis, which may lead to disruption of mitochondrial permeability transition pore and eventually cell death (Bezprozvanny and Mattson, 2008; Reddy, 2009). Insulin has also been described to impact intracellular calcium homeostasis. A recent study by the Thibault lab reported that acute insulin administration decreases calcium transients ultimately affecting the function of intracellular calcium channels (Maimaiti et al., 2017). Understanding this relationship is necessary, as higher levels of intracellular calcium may consequently disrupt physiologic glucose metabolism in the brain; potentially promoting further pathology (Pancani et al., 2011).

Epidemiological evidence has described parental history of AD as being an important predisposition to developing the disease (Fratiglioni et al., 1993). The risk may be more prominent for maternal than paternal history and lead to AD-related brain changes, even in cognitively normal adults (Edland et al., 1996; Mosconi et al., 2007, 2009a). As mitochondrial DNA (mtDNA) is inherited from the mother, any maternal genetic predispositions for bioenergetic disturbances may be passed down leading to increased risk for disease (Taylor and Turnbull, 2005).

Even prior to the first articles concerning an Amyloid Cascade Hypothesis of AD (Hardy and Higgins, 1992), evidence described mitochondrial/bioenergetic disturbances in AD (Sims et al., 1987; Parker et al., 1990; Blass and Gibson, 1991). Blass (2000) described a Mitochondrial Spiral as contributing significantly to AD pathogenesis. Three main points were discussed: reduced brain (glucose) metabolism, oxidative stress and calcium dysregulation. Deficiencies in either of these domains may ultimately led to disruption in the others—contributing to the pathogenesis of AD (Blass, 2000). Swerdlow and Khan (2004) proposed a Mitochondrial Cascade Hypothesis of sporadic AD. This theory of AD pathogenesis identifies the mitochondria and mitochondrial dysfunction as a central mediator in the development of LOAD. The inheritance of genes important for bioenergetic processes combined with various environmental influences throughout life could lead to the pathological changes associated with AD. One such environmental influence is insulin resistance. The impaired energy metabolism seen in insulin resistance may catalyze the bioenergetic deficits that contribute to the mitochondrial dysfunction seen in AD (Correia et al., 2012; Montgomery and Turner, 2015). Disrupted insulin signaling leads to impaired energy metabolism, likely affecting the bioenergetic machinery working to maintain an adequate supply of energy for bodily function. There is still debate whether insulin resistance leads to mitochondrial dysfunction, if mitochondrial dysfunction contributes to insulin resistance, or if they mutually impact each other (Montgomery and Turner, 2015). Regardless of causality, mitochondrial dysfunction is fundamental feature of insulin resistance. Thus, insulin resistance may either promote or intensify the bioenergetic dysfunction already apparent in AD.

## Cerebral Metabolism and The Bioenergetic Shift in Alzheimer’s Disease

Given the systemic metabolic dysfunction seen in AD potentially promoted by insulin resistance, it is important to understand the various metabolic fuels that may be used by the brain and how our current knowledge of brain metabolism is limited by the study of mainly one of these fuels. In this section, we will also discuss a potential bioenergetic shift in brain metabolism in AD and how this may be visualized with neuroimaging techniques prior to significant disease pathology.

### Metabolic Fuels

Several sources of energy may be utilized in the production of adenosine triphosphate (ATP) that is required for many physiologic processes (Wallace et al., 2010). It is well established that glucose is the primary source of brain metabolic energy for healthy individuals. The interaction between neurons and glia, especially astrocytes, is fundamental for brain energy metabolism (Pellerin et al., 2007; Bélanger et al., 2011); and must be appreciated when interpreting results of cerebral metabolic studies. Glucose may be metabolized into pyruvate and lactate, via erobic and anerobic glycolysis, both of which have received attention concerning their roles in brain metabolism (Gonzalez et al., 2005; Boumezbeur et al., 2010; Barros, 2013). Furthermore, accumulating evidence describes the importance of erobic glycolysis in brain function (Vaishnavi et al., 2010; Vlassenko et al., 2010).

When glucose is in excess it is stored as glycogen, which is largely stored in the liver for systemic use and in the skeletal and cardiac muscle for local utilization (Shulman et al., 1990; Roden et al., 1996). However, glycogen has also been found within brain astrocytes (Brown and Ransom, 2007). There is debate to how long these stores may provide energy for the brain. Yet, astrocytic stores likely make glycogen an important source of cerebral fuel in times of need.

In addition to glucose and its derivatives, other sources may be used as metabolic fuels. Beta-oxidation of fatty acids may be a significant source of fuel for systemic energy metabolism, especially at times when glucose and glycogen reserves are depleted or within states of diminished glucose metabolism (Cahill, 2006). Even though fatty acids may cross the BBB, they are not the preferred alternate substrate to glucose for brain metabolism. Schonfeld and Reiser suggest that fatty acids may be less efficient with a slower rate of oxidation than glucose and ketone bodies, while being associated with higher rates of oxidative stress (Schönfeld and Reiser, 2013).

Ketone bodies (acetoacetate (AcAc), beta-hydroxybutyrate (BHB) and acetone) or KB are mainly synthesized in the liver as a result of beta-oxidation of fatty acids and are the primary alternative fuel to glucose in brain metabolism (Garber et al., 1974). They are released in marginal amounts in healthy individuals, and in times of fast or on a high fat ketogenic diet levels are increased compensating for decreased glucose metabolism (Cahill, 2006). Ketone bodies are more efficient than glucose relative to the amount of oxygen needed to carry out oxidative metabolism (Cahill, 2006). The lower amount of oxygen required for KB metabolism not only conserves the use of a vital resource (especially in hypoxic states), but it leads to decreased generation of ROS that may promote cellular damage and eventual cell death. Furthermore, increased KB metabolism may lead to an improved glucose metabolism, which may add to the efficacy of a therapy aimed at ketosis (Roy et al., 2012). In states where circulating KB are elevated, transporters regulating their flux into the BBB are upregulated. These monocarboxylate transporters (MCTs) are known to transport various substrates depending on their class, including: KB, lactate, pyruvate and thyroid hormone (Halestrap and Wilson, 2012).

In the brain, MCTs are found in the cell membranes of neurons and glia in addition to the BBB. Even though KB only marginally contribute to brain metabolism under basal conditions in a healthy individual, they may constitute over 60% of brain energy metabolism during the fasted state, and help mitigate decreased glucose metabolism (Morris, 2005). There are three main determinants of KB use by the brain: concentration of KB in the blood, transport across the BBB via MCTs, and cerebral activity of enzymes used in KB metabolism (Morris, 2005). KBs become a significant source of metabolic fuel as their plasma concentration increases. After transport across the BBB, research suggests that the rate-limiting factor in the utilization of KB is activity of catalytic enzymes that may regionally vary throughout the brain (Morris, 2005). However, other research describes the rate-limiting step after acute KB administration as transport across the BBB (Blomqvist et al., 2002). MCTs on the BBB are upregulated in states of chronic ketosis (Halestrap and Wilson, 2012). With an acute administration of KB, MCT density on the BBB may be the rate-limiting step for the ability of KB to be used in brain metabolism, while in a chronic state of ketosis the environment is adapted to a state of decreased glucose metabolism and may be dependent on the ketolytic metabolic machinery.

### Brain Hypermetabolism in Alzheimer’s Disease and Related Disorders

One of the most prominent features of the underlying mitochondrial and metabolic abnormalities in AD may be brain glucose hypometabolism. Disrupted cerebral glucose metabolism is visible using FDG PET and one of the earliest pathologic events in AD (Mosconi, 2005; Mosconi et al., 2008; Sperling et al., 2011). The hypometabolic pattern seen in AD is fairly characteristic. Metabolic declines are first found in the parietal-temporal area, posterior cingulate cortices and medial temporal lobes. Hypometabolism eventually progresses to the frontal lobes, subcortical areas and the cerebellum (Mosconi et al., 2009b). This may occur more than a decade prior to clinical onset of AD and is most evident in individuals that carry an APOE4 allele or with a positive family history (especially maternal history) of dementia (Mosconi et al., 2009b; Cunnane et al., 2011; Sperling et al., 2011). However, in recent years mounting evidence has described a state of reactive or compensatory glucose hypermetabolism in AD as well as Parkinson’s, Huntington’s and other neurologic diseases (Gilman et al., 1990; Haier et al., 2003; Borghammer et al., 2012; Cistaro et al., 2012; Lee et al., 2012; Ashraf et al., 2015).

Ashraf et al. (2015) reported cortical glucose hypermetabolism in individuals with amnestic MCI. Glucose hypermetabolism was found in amyloid (11C-PiB PET) negative (*n* = 4) and amyloid positive (*n* = 1) participants. Opposing results were seen in participants with the highest amyloid load (*n* = 5), which were found to have cortical glucose hypometabolism. Interestingly, amyloid negative participants with glucose hypermetabolism did not convert to AD within an 18-month follow-up, while amyloid positive participants (*n* = 4) did convert to AD within the same time period (Ashraf et al., 2015). Results from this study suggest that cerebral glucose hypermetabolism may precede glucose hypometabolism in the earliest stages of AD pathology.

Findings of compensatory hypermetabolism in a clinical population with MCI/AD have been supported by evidence describing a similar cerebral metabolic profile in AD mouse models. In the APP/PS1 mouse model of AD, Poisnel et al. (2012) found cortical and hippocampal glucose hypermetabolism in APP/PS1 mice relative to controls at 12 months. Moreover, the group used autoradiography to show that the increased glucose uptake was generally limited to areas near amyloid plaque accumulation (Poisnel et al., 2012). Similar results have been found in the 5xFAD (Rojas et al., 2013) and Tg2576 mouse models of AD (Luo et al., 2012).

Cerebral glucose hypermetabolism has also been described in several other neurologic disorders, commonly preceding a state of impaired glucose metabolism in a disease-specific manner. In Huntington’s disease (HD), relative cerebral glucose hypermetabolism has been described in several brain areas including the thalamus, while hypometabolism was seen in the striatum (Lee et al., 2012). In Parkinson’s disease (PD), hypermetabolism has been found in the globus pallidus externus and other subcortical areas. This contrasts the near-global cortical hypometabolism seen in PD (Borghammer et al., 2012). Patients with bulbar and spinal-onset Amyotrophic Lateral Sclerosis (ALS) had higher glucose metabolism on FDG PET than controls in several regions of interest (ROIs), including the amygdala, midbrain, pons, and cerebellum. While lower glucose metabolism was found in frontal/parietal lobes in those with bulbar-onset alS relative to spinal-onset patients and controls (Cistaro et al., 2012). In Down Syndrome, inferior temporal lobe and entorhinal cortex hypermetabolism has been reported prior to the onset of dementia, which may be a compensatory response to early pathologic changes (Haier et al., 2003, 2008). Similar evidence has been found in the study of Friedreich’s ataxia, where glucose hypermetabolism was reported in those with early disease. An apparent glucose hypometabolic state was associated with further progression of the clinical condition. This difference between hypermetabolic to hypometabolic state was noted by authors to be regionally specific (Gilman et al., 1990). In theory, similar metabolic changes could occur in a disease-specific pattern in any disorder of the CNS with significant neurodegenerative pathology.

Intriguingly, cerebral energy metabolism may be impacted by systemic metabolic disease without clinically defined neuropathology. In Willette et al. (2015a) examined the relationship of systemic insulin resistance (HOMA-IR) and cerebral glucose metabolism. They found a higher degree of insulin resistance was related to lower glucose metabolism in AD ROIs. Conversely, in participants with MCI that eventually progressed to AD, higher HOMA-IR was associated with glucose hypermetabolism in the medial temporal lobes and hippocampus (Willette et al., 2015a,b). These results suggest that there may be a differential cerebral metabolic response throughout the clinical spectrum of AD and highlight the importance of studying the relation of brain metabolism to systemic metabolic risk factors. Moreover, our lab has previously reported that older adults with insulin resistance showed AD-like patterns of reduced brain glucose metabolism, as quantified with FDG PET imaging. Glucose hypometabolism was most apparent in frontal, parietotemporal, and cingulate cortices (Baker et al., 2011). This suggests that insulin resistance may promote AD-related changes in brain in those at-risk for the disorder.

Although underlying synaptic dysfunction and neuronal degeneration likely promotes decreased glucose utilization in the brain (cerebral glucose hypometabolism on FDG PET imaging) there is still debate as to whether an inherent glucose hypometabolism causes the early pathologic changes seen in AD and related disorders. A review by Cunnane et al. (2011) explores this topic by offering several mechanisms by which glucose hypometabolism may contribute to AD-related pathology. Potential mechanisms described include tau hyperphosphorylation resulting from lower glucose availability, decreased glycolytic enzyme activity—with a potential impact on cholinergic neurotransmission, and brain microvascular changes that may disrupt glucose influx into the brain (Cunnane et al., 2011). Yet, another potential mediator of the AD-related brain glucose changes may be a bioenergetic shift in metabolism from glucose-based to KB-based.

### Brain Bioenergetic Shifts and Alzheimer’s Disease Pathogenesis

The notion of a bioenergetic shift in energy metabolism in AD has been described in the literature spurred by observations concerning the metabolic deficits commonly seen in the disorder—especially glucose hypometabolism, mitochondrial dysfunction and oxidative stress (Kadish et al., 2009; Yao et al., 2011). Important work has come from Roberta Brinton and colleagues, who have described brain changes resulting from a potential bioenergetic shift, with special interest in how this shift is impacted by estrogen (Brinton, 2008a,b; Yao et al., 2009, 2010, 2011, 2012; Yao and Brinton, 2012; Ding et al., 2013a,b). Evidence using a female triple transgenic AD mouse model (3xTg-AD) and non-transgenic mice describes an upregulation of succinyl-CoA:3-ketoacid coenzyme A transferase (SCOT) and hydroxyacyl-coenzyme A dehydrogenase (HADH) during reproductive senescence (Yao et al., 2010). SCOT is an important enzyme for KB energy metabolism, while HADH is an enzyme with a role in the beta-oxidation of fatty acids (Fukao et al., 1997; Houten and Wanders, 2010). Notably, this upregulation of SCOT and HADH occurred concurrent with the decreased activity of PDH, an important enzyme in glucose energy metabolism (Yao et al., 2010). When coupled with an apparent inefficiency of glucose metabolism in AD, an upregulation of genes involved in KB (and fatty acid) metabolism strongly supports a bioenergetic shift phenomenon.

In a state where glucose metabolism is disrupted, it would be beneficial to increase the fraction of metabolism that is contributed by an alternate source of fuel. Genes involved in KB and fatty acid metabolism would be upregulated, while genes driving glycolytic metabolism may be downregulated. However, even with a change in metabolic machinery favoring KB and fatty acid metabolism, if the newly preferred metabolic fuel is not supplied through diet then it must come another source. When exogenous glucose supply is low in a healthy adult, glucose is derived from the breakdown of stored glycogen and through gluconeogenesis (Cahill, 2006). If glycogen stores are depleted and the amount of gluconeogenesis is not sufficient to meet energy demands, then the use of fats, KB, and even amino acids (in later stages) are utilized as fuel by the body (Cahill, 2006). If an individual is progressing through a systemic bioenergetic shift in which the preferred brain fuel substrate are KB and there is an inadequate supply of this fuel through exogenous intake, then the fuel must come from an endogenous source. Adipose tissue reserves are generally sizeable enough to supply fuel through beta-oxidation of fatty acids, leading to the production of KB that are largely produced by the liver (Garber et al., 1974). Ketone bodies can then be transported to the brain across the BBB for use in the production of ATP (Halestrap and Wilson, 2012). As circulating levels of KB become elevated brain usage increases; with capacity for utilization limited by expression of ketolytic enzymes (Morris, 2005). Over time, however, the capacity to compensate with peripheral KB production may diminish without proper exogenous supply through diet or supplementation (Yao et al., 2011). This could lead to a state where the brain becomes dependent on itself as a source of fatty acids and KB.

The myelin insulating neuronal axons in the brain is largely made of various lipids, cholesterol and proteins (Quarles et al., 2006). Thus, it may be a ready source of the fatty acid fuel that may be metabolized into KB for use by the brain. Phospholipase-A2 (PLA2) is an enzyme that catalyzes the cleavage of fatty acids from phospholipids (mainly in cellular membranes) as well as the release of arachidonic acid (AA) and eicosanoid synthesis (Sun et al., 2004; Adibhatla and Hatcher, 2008). When PLA2 is activated in the brain, it may lead to the breakdown of myelin (Adibhatla and Hatcher, 2008), releasing fatty acids for potential use as fuel with ultimate conversion to KB with interaction between astrocytes and neurons. An increased production of ROS, such as hydrogen peroxide, seen in mitochondrial dysfunction also leads to an activation of PLA2 provoking further myelin breakdown (Adibhatla and Hatcher, 2008; Sun et al., 2010; Yao et al., 2011). As the myelin is degraded, white matter changes may become apparent through various neuroimaging modalities and over time may contribute to gross volumetric loss and cognitive changes. White matter abnormalities are a common pathology seen as early as preclinical AD (Burns et al., 2005; Medina et al., 2006). Furthermore, PLA2 has been associated with the pathological response associated with Aβ (Zhu et al., 2006) and has been shown to be increased in the AD brain (Sanchez-Mejia et al., 2008). Work by Sanchez-Mejia et al. (2008) showed that a reduction in PLA2 activation diminished the PLA2-associated neurotoxicity and learning/memory deficits. Thus, the underlying AD-related Aβ accumulation, mitochondrial dysfunction, and bioenergetic shift may each contribute to the activation of PLA2 and ultimately lead to characteristic pathology of the disorder. Yet, it remains to be determined to what extent each of the mediators of PLA2 activation is the primary contributor.

Additional evidence of a bioenergetic shift in AD is the weight changes that are seen in the disorder (White et al., 1998). Individuals prior to AD diagnosis on average tend to weigh more than the general population. Starting with preclinical disease and progression to MCI and Alzheimer’s dementia there seems to be a significant weight loss with each stage of decline (White et al., 1998; Johnson et al., 2006). This may be due to a combination of factors, such as decreased appetite, co-morbid depression, and impairments in various cognitive functions (Gillette-Guyonnet et al., 2000). Yet, these weight changes may also relate to the bioenergetic shift in energy metabolism.

**Figure 1:**
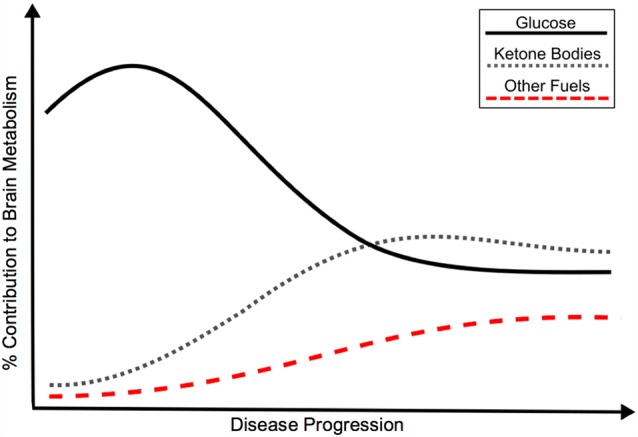
Hypothetical model of the bioenergetic shift in energy metabolism in Alzheimer’s disease (AD), as represented by relative contribution of glucose, ketone bodies and other fuels to brain metabolism. Although classically associated with glucose hypometabolism, recent evidence points to an initial glucose hypermetabolic state with potential shift towards use of alternate fuels. Insulin resistance may promote the development of AD through a variety of mechanisms, including a shift in energy metabolism. The bioenergetic shift may also be an independent feature of AD pathology. Black: Glucose metabolism; Gray: Ketone body metabolism; Red: Other fuel metabolism.

Given the current evidence and need to understand AD pathologic processes, we support an updated model for cerebral metabolic changes in AD. The ultimate endpoint of brain energy utilization in AD and many other disorders is hypometabolism, however, a hypermetabolic glucose compensatory response may result from pathology early in disease progression (pre-clinical stage). With increased demand for energy and/or decreasing efficiency of glucose metabolism, alternate fuels (i.e., ketone bodies) may also supplement glucose metabolism. With worsening pathology and significant cell death, global hypometabolism develops. It is likely that these CNS metabolic changes may be provoked by systemic disease. Figure 1 displays a representation of the metabolic transitions we hypothesize are seen throughout the continuum of sporadic AD.

If a metabolic bioenergetic shift is indeed a feature of AD, several key questions remain to be answered. What causes this shift? Is it an inherent defect in mitochondrial bioenergetics or a result of amyloid, tau, and other AD-related pathology? How do diseases of systemic metabolic dysfunction (insulin resistance, hyperlipidemia, etc.) impact the development of a bioenergetic shift and how does this relate to the susceptibility to develop AD? Further research into the role of metabolic dysfunction and mitochondrial bioenergetics in AD may answer these questions and ultimately fortify our understanding of AD pathogenesis.

### Neuroimaging Biomarkers of Bioenergetic Shift in Alzheimer’s Disease

Our ability to study the bioenergetic changes in AD is enhanced by newly developed imaging techniques, which may ultimately lead to the identification of a biomarker at the earliest signs of metabolic disturbance in AD and a way to quantify changes secondary to therapeutic intervention.

Although functional imaging of glucose metabolism via FDG PET is commonly used in research and clinical settings, solely visualizing brain glucose metabolism does not provide a comprehensive understanding of the brain’s differential use of fuels. In order to appreciate the bioenergetic shift that potentially contributes to the pathogenesis of AD, it is important to study the use of KB, as they are the primary alternate fuel for brain metabolism. Several neuroimaging methods may be utilized for the study of brain KB metabolism, including PET with tracers developed for individual KB and magnetic resonance spectroscopy (MRS; Blomqvist et al., 1995, 2002; Pan et al., 2000; Tremblay et al., 2007, 2008).

Literature regarding the utilization of PET imaging for cerebral alternate energy metabolism is relatively scarce, largely originating from two main laboratories. Work from Blomqvist et al. (1995, 2002) described the use of carbon-11 labeled BHB PET imaging. BHB is one of the KB utilized by the brain and can be readily measured peripherally in the blood (Garber et al., 1974). Studies using this tracer described a BBB transport-dependent uptake of BHB after acute infusion of the KB (Blomqvist et al., 1995, 2002). Findings suggested no significant difference in uptake or utilization of BHB in metabolically healthy subjects and those who had insulin dependent (type 1) diabetes (Blomqvist et al., 2002). An earlier study by the group using the same tracer described BHB utilization increasing linearly with peripheral concentration and showed higher BHB utilization in gray over white matter (Blomqvist et al., 1995). These results were important in establishing a PET technique that may be utilized to visualize differential brain metabolism. Moreover, they provide valuable evidence to how KB utilization changes with peripheral concentration.

More recently, Cunnane and colleagues have developed a carbon-11 labeled radiotracer of AcAc, the other major KB utilized by the brain, for use in PET imaging of brain KB metabolism (Tremblay et al., 2007). Their work describes use of the tracer in rat models of aging and on ketogenic interventions and clinical studies. Results suggest an increased KB uptake in young and aged rats during a ketogenic intervention (Bentourkia et al., 2009; Roy et al., 2012). Importantly elevated cerebral 11C-AcAc uptake corresponds to amount of peripheral KB levels (Bentourkia et al., 2009; Roy et al., 2012). Although the use of an AcAc tracer is exciting and informative, the Cunnane group has implemented a novel dual-tracer PET approach with 11C-AcAc and 18F-FDG at the same imaging session. Several articles in the human population and within rodent models have utilized this dual-tracer technique (Roy et al., 2012; Nugent et al., 2014; Castellano et al., 2015). In a comparison of healthy young (mean age = 26 years) and older adults (mean age = 74 years), both glucose and AcAc metabolism was decreased in older adults relative to the younger cohort (Nugent et al., 2014). A 2015 study has explored cerebral glucose and AcAc metabolism in AD (Castellano et al., 2015). They described a reduction in cerebral glucose metabolism in older adults with mild AD relative to age-matched controls. Interestingly, AcAc did not differ between the groups (Castellano et al., 2015), which may provide a therapeutic outlet for those with AD and related disorders. This dual-tracer technique has the potential to greatly expand our understanding of brain metabolism in healthy vs. pathological conditions, how it is affected in “normal aging” as well as in AD. There is much to be gained from the study of brain KB metabolism, especially when coupled with glucose metabolism. The ability to visualize the bioenergetic shift in energy metabolism may provide further insight into the development of AD and ultimately act as an early biomarker for AD and the ability to determine individuals that may benefit from therapies aimed at providing KB as an alternative source of fuel for therapeutic treatment.

## Conclusion

In this review, we have discussed the contribution of insulin resistance to the pathogenesis of AD, in particular how insulin resistance may induce a bioenergetic shift in peripheral and CNS energy metabolism, while closing with a review of neuroimaging biomarkers that may be used to identify and better understand the bioenergetic changes seen in AD and potentially response to preventative and therapeutic interventions.

We remain at a time where we have made significant progress in understanding AD without any disease-modifying therapeutics or proven prevention strategies. Yet, we now have the opportunity to explore new areas of research that may expand our knowledgebase and provide a more comprehensive view of AD pathogenesis. One of the most vital areas of need is the study of brain metabolism. Until now we have largely discussed glucose as being the sole player in brain metabolism. In fact, when brain energy is discussed, it is implied that we are referring to glucose metabolism (i.e., cerebral [glucose] metabolism). We take for granted our knowledge that glucose is the primary fuel—that higher uptake is better and that in almost every neurologic and psychiatric condition, glucose metabolism is diminished (Mosconi et al., 2009b; Wallace et al., 2010; Bélanger et al., 2011; Bohnen et al., 2012). Studies describing a state of compensatory or reactive glucose hypermetabolism that precedes significant clinical decline are overshadowed by reports of the hypometabolic state commonly assumed in most neurologic disorders (Bohnen et al., 2012; Borghammer et al., 2012; Cistaro et al., 2012; Lee et al., 2012; Ashraf et al., 2015). Although the cause of this hypermetabolism is not definitively known, it is most likely a compensatory response to injury and initial pathologic processes (Ashraf et al., 2015). If this was an isolated phenomenon, it would be easy to dismiss. However, it has been reported in conditions from Alzheimer’s, Parkinson’s, Huntington’s and ALS, to Down Syndrome, Friedrich’s Ataxia and familial Creutzfeldt-Jakob disease in disease-specific patterns (Gilman et al., 1990; Haier et al., 2003; Nagasaka et al., 2011; Borghammer et al., 2012; Cistaro et al., 2012; Lee et al., 2012; Ashraf et al., 2015).

The view of a glucose hypermetabolic state as an early disease event and response to initial pathology is likely a temporary solution to injury with an ultimate decline to decreased glucose utilization. If further evidence supports the occurrence of an initial rise and ultimate decline of cerebral glucose metabolism, then it possible to visualize this shift early (prior to clinical symptomology) and work to prevent the underlying pathology. This primary glucose hypermetabolic shift is likely also supplemented with a increase in utilization of other fuels. With progression of the disease, a potential bioenergetic shift may occur with decreasing reliance on glucose and increased use of alternate energy sources, see Figure 1.

Despite the brain’s metabolic flexibility, most alternate fuels have been inadequately studied. These are sources of energy that our bodies use on a daily basis. Ketone bodies are an optimal first alternate fuel to study as they have been commonly reported to be the chief alternate source of cerebral energy (Cahill, 2006). Yet, acetate, lactate, pyruvate, amino acids or even glycogen remain largely unstudied (Gonzalez et al., 2005; Boumezbeur et al., 2010; Wallace et al., 2010; Barros, 2013; Cunnane et al., 2016). It is crucial to understand the brain’s use of different fuels and how this may change throughout progression of disease. This enables us to appreciate pathophysiology and the body’s response to increasing pathology. Moreover, we may be able to characterize “brain metabolic fingerprints” that may be used to offer patients and research participants more personalized therapeutic options if they were to develop a condition impacting brain metabolism. Strategies for prevention and therapeutic approaches may be developed to improve metabolism and clinical function. Importantly, due to the metabolic changes commonly seen in brain disease, any findings from the study of one disorder may have broad application to other neurologic, psychiatric, as well as systemic metabolic conditions.

Studying the brain in any context is truly a new frontier, and we have only recently had the opportunity and tools to study its majesty. By investigating areas such as differential brain energy metabolism, we have the opportunity to greatly advance our understanding of how our brains function in health and disease. Many mysteries and questions remain—and if answered may lead to the successful treatment and prevention of some of the most terrifying diseases of our time.

## Author Contributions

BJN and SC drafted, revised and approved final version of the manuscript.

## Conflict of Interest Statement

The authors declare that the research was conducted in the absence of any commercial or financial relationships that could be construed as a potential conflict of interest. Parts of this manuscript are derived from unpublished material of author’s (BJN) PhD dissertation. The reviewer OT and handling Editor declared their shared affiliation.
